# Follicular Lymphoma Diagnosed Successfully via Endobronchial Ultrasound-Guided Intranodal Forceps Biopsy Located in Left Lymph Node #12

**DOI:** 10.7759/cureus.73486

**Published:** 2024-11-11

**Authors:** Suzuka Matsuoka, Yuki Takigawa, Ken Sato, Akiko Sato, Tetsuya Isoda

**Affiliations:** 1 Department of Respiratory Medicine, National Hospital Organization Okayama Medical Center, Okayama, JPN; 2 Department of Respiratory Medicine, Okayama Medical Center, Okayama, JPN; 3 Department of Pathology, Okayama University Hospital, Okayama, JPN

**Keywords:** bronchoscopy, ebus-tbna (endobronchial ultrasound-transbronchial needle aspiration), endobronchial ultrasound-guided intranodal forceps biopsy, follicular lymphoma, lymph-node

## Abstract

A 71-year-old man with follicular lymphoma of the right inguinal lymph node was referred to our hospital owing to mediastinal lymph node enlargement (left #12). The patient had a history of cyclosporine (CYS-A) and steroid therapy for fibrotic hypersensitivity pneumonitis. Endobronchial ultrasound-transbronchial aspiration and endobronchial ultrasound-guided intranodal forceps biopsy (EBUS-IFB) were performed under conscious sedation using midazolam and fentanyl. Histological examination of the tissue obtained by EBUS-IFB soley revealed follicular lymphoma.

## Introduction

Regarding the diagnosis of isolated mediastinal lymphadenopathy, which is suspected of lymphoma, minimally invasive endoscopic diagnostic modalities, such as endobronchial ultrasound-transbronchial aspiration (EBUS-TBNA) [1] and endoscopic ultrasound-fine needle aspiration [2], have been commonly used. However, tissues obtained using EBUS-TBNA are sometimes considered unsatisfactory for diagnosis owing to their small size or quality. EBUS-TBNA, followed by additional EBUS-guided intranodal forceps biopsy (EBUS-IFB), is expected to increase the diagnostic yield [3]. EBUS-IFB, which is a novel method for bronchoscopic biopsy of mediastinal lymph nodes, involves the passage of mini forceps into targeted lymph nodes through the EBUS-TBNA puncture tract.

## Case presentation

**Figure 1:**
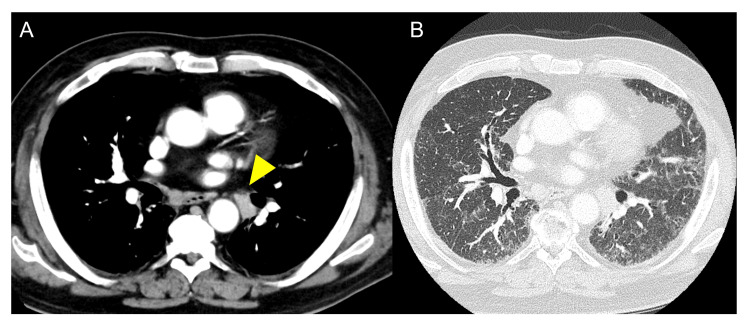
Image of the first admission to our institution. (A) and (B) Contrast-enhanced computed tomography on admission showing enlarged lymph nodes (yellow arrow).

A 71-year-old man was diagnosed with fibrotic hypersensitivity pneumonitis (fHP), and the patient had been treated with prednisolone since 2018. Four years after diagnosis, cyclosporine (CYS-A) (100 mg/day) was added and increased to 200 mg/day. One year after the start of CYS-A, chest computed tomography (CT) revealed enlargement of the right inguinal lymph node with no other abnormalities. Following a surgical biopsy performed under local anesthesia, the patient was diagnosed with follicular lymphoma arising in immune deficiency. CYS-A was discontinued, and curative radiotherapy was performed on the malignant lymph nodes in the right inguinal region at the previous hospital. During outpatient follow-up, positron emission tomography revealed abnormal fluorodeoxyglucose uptake in the left hilar lymph node (left #12). The serum tumor markers were as follows: carcinoembryonic antigen (CEA) = 8.4 ng/mL (normal range, <5.0 ng/mL), cytokeratin fragment (CYFRA) = 5.8 ng/mL (normal range, <2.0 ng/mL), and interleukin-2 (IL-2) receptor = 445.0 U/mL (normal range, <496.0 U/mL). These findings were indicative of a new primary lung cancer or lymphoma involvement; therefore, the patient was referred to our respiratory medicine department for lymph node evaluation. CT revealed enlarged lymph nodes (14 mm) in the left hilum (Figure 1A) and no pulmonary nodules. Interstitial changes caused by fHP were also observed (Figure 1B).

**Figure 2:**
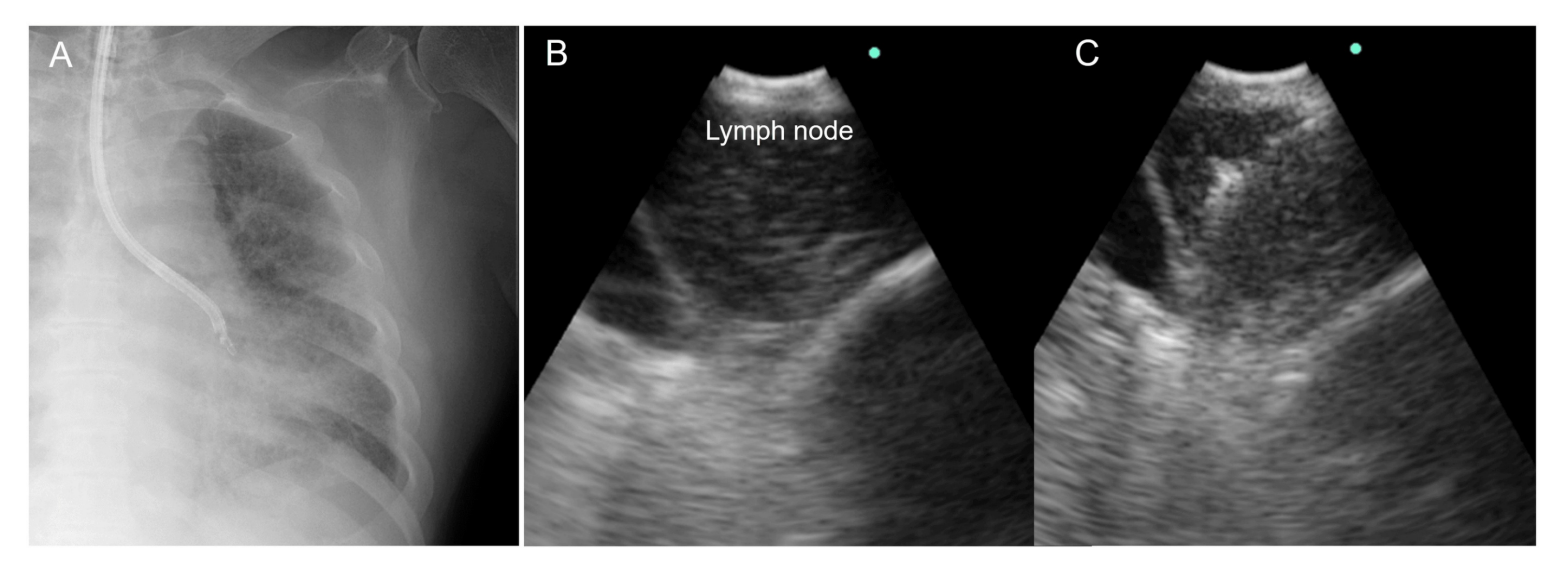
Endobronchial ultrasound images. (A) X-ray shows a convex probe adjacent to the target lesion. (B) An endobronchial ultrasound (EBUS) image confirming an enlarged #12 lymph node. (C) The mini-forceps were inserted into the left #12 lymph node, and an opening in the lymph node was confirmed.

Bronchoscopy was performed under local anesthesia and conscious sedation using midazolam and fentanyl. A convex EBUS bronchoscope (BF-UC290F; Olympus, Tokyo, Japan) was advanced into the left lower lobe (Figure 2A) and confirmed to be adjacent to the lesion using a convex EBUS probe (Figure 2B). Four specimens were obtained from the target lymph nodes using a 21-gauge needle (ViziShot2, Olympus, Center Valley), and rapid on-site evaluation yielded nonspecific results. Therefore, EBUS-IFB was performed with mini-forceps (CoreDx^TM^, Boston Scientific) to obtain high-quality core tissue. EBUS-IFB and mini-forceps biopsy were performed four times (Figure 2C). The rapid on-site evaluation was positive for all the IFB tissues suspected of lymphoma. The total examination time for EBUS-TBNA and EBUS-IFB was 26 min (16 min for EBUS-TBNA and 10 min for EBUS-IFB). Bleeding and other complications, such as pneumomediastinum, pneumothorax, and acute exacerbation, did not occur. The maximum size of the specimens obtained using EBUS-IFB was 3.76 mm^2^.

**Figure 3:**
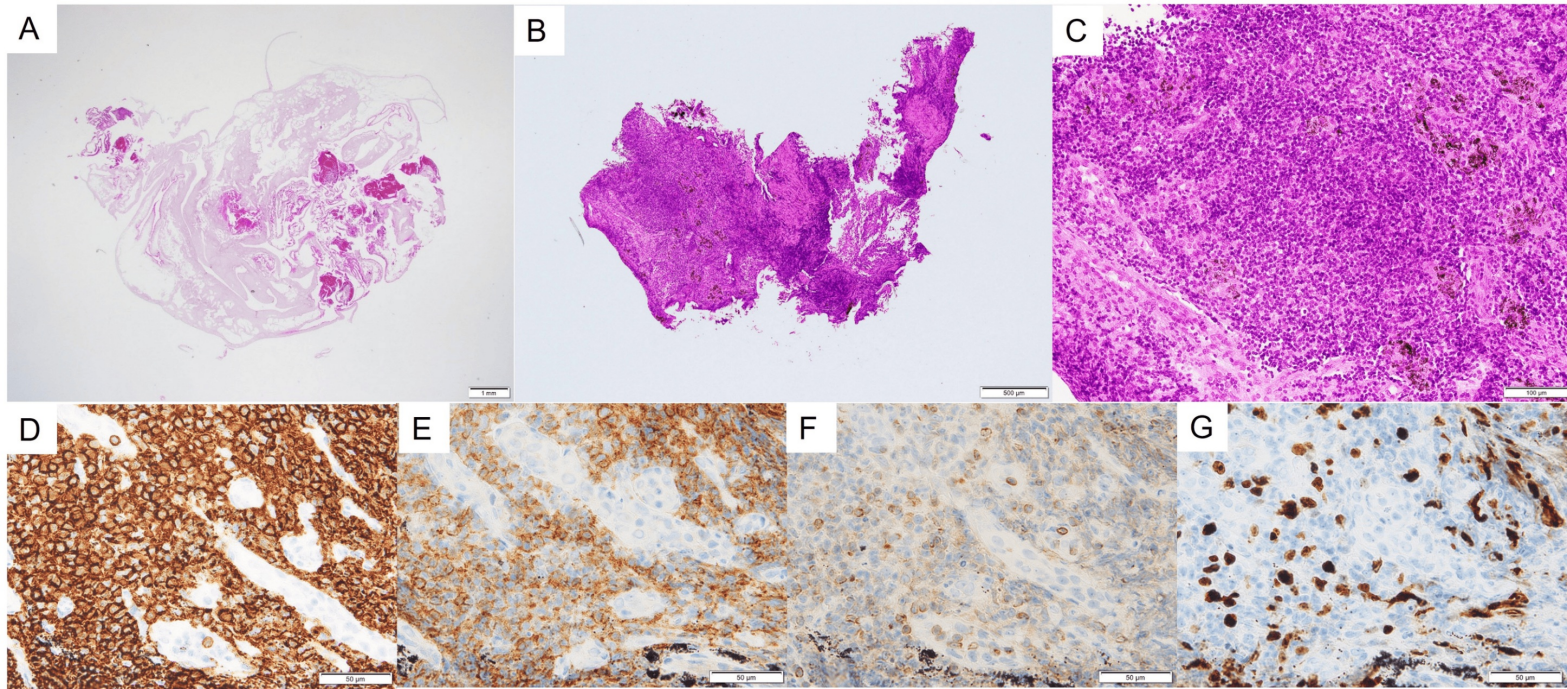
Histological findings. (A) Tissue obtained using endobronchial ultrasound-transbronchial aspiration (×40). (B) and (C) Tissue obtained using EBUS-intranodal forceps biopsy (EBUS-IFB) (×40, ×200). Immunohistochemical staining: (D) CD20-positive, (E) CD10-positive, (F) BCL2-weakly positive, and (G) Ki-67 proliferation index-low.

Compared to the pathological findings of the specimens obtained via EBUS-TBNA (Figure 3A), which showed only a few lymphoid cells, the specimens obtained via EBUS-IFB revealed infiltration of small-to-medium-sized lymphoid cells (Figures 3B, 3C). The tumor cells were CD20-positive, CD10-positive, BCL2-weakly positive, and TdT-negative, and the Ki-67 proliferation index was low (Figures 3D-3G). Flow cytometry was positive for CD19, CD20, CD22, and CD79a. CD10 and CD23, which are characteristic of follicular lymphoma, were also positive. The histopathologic findings obtained by EBUS-IFB were consistent with those of the inguinal lymph node tissue. Therefore, the patient was diagnosed with a recurrence of follicular lymphoma.

## Discussion

The diagnostic yield of EBUS-TBNA for the evaluation of lymphoma is lower than that for lung cancer. EBUS-TBNA is an adequate option for diagnosing lung cancer, with a sensitivity of approximately 95% [4]. On the other hand, the sensitivity for the deﬁnitive diagnosis of lymphoma was 57% because of the difficulties of confirming the diagnosis of lymphoma on a small volume specimen in some disease subtypes, such as marginal zone lymphoma, follicular lymphoma, or Hodgkin lymphoma (HL) [5]. Diagnosis of lymphoma requires a large sample due to evaluation of cytology, immunophenotype, and histology. Samples of tissues are also needed for molecular analysis via G-banding and immunophenotyping via flow cytometry. Non-Hodgkin lymphoma (NHL) and HL treatment depend on specific subtyping and histologic grade. Therefore, some patients require further surgical biopsy to evaluate cell morphology, immunophenotype, and histology [5]. Grosu et al. reported the sensitivity of EBUS-TBNA for de novo lymphoma was 67%, and the sensitivity for recurrence lymphoma was 81% [6]. The diagnosis of de novo lymphoma is more difficult for diagnosis than that of recurrence lymphoma.

**Figure 4:**
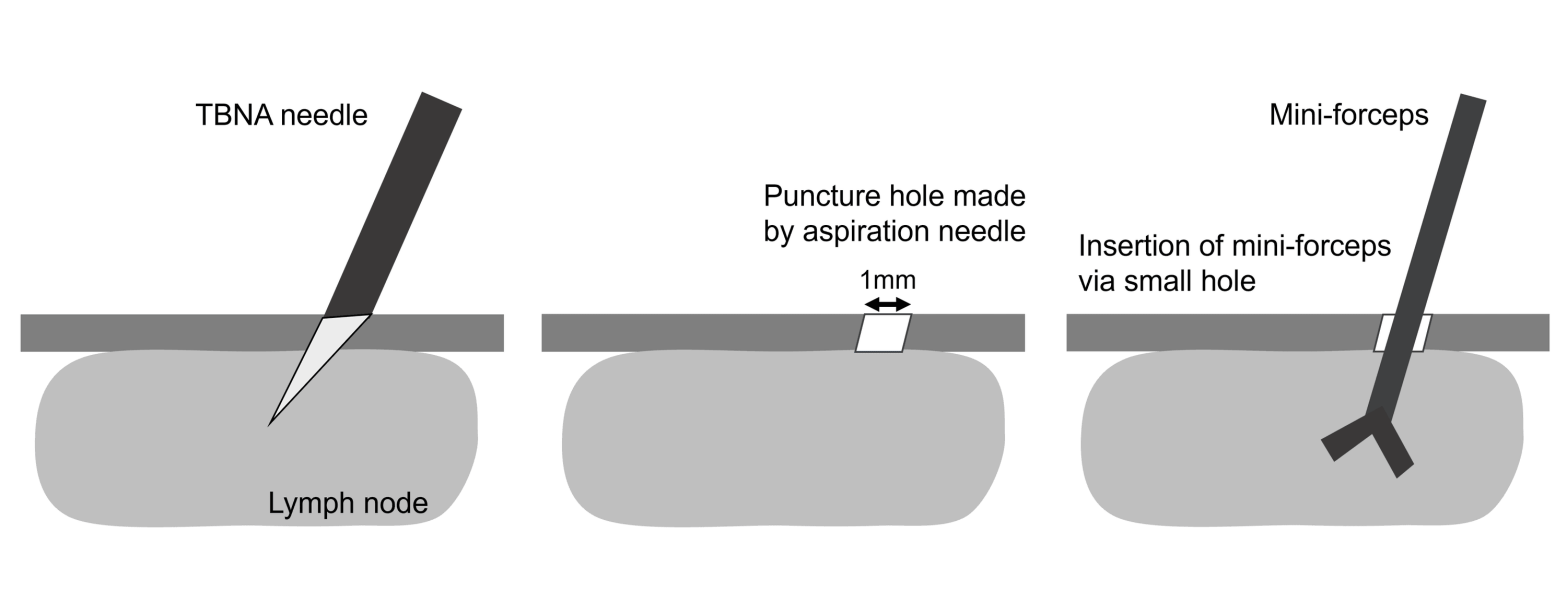
Endobronchial ultrasound-intranodal forceps biopsy (EBUS-IFB) procedure. Aspiration creates a hole of more than 1 mm in the airway mucosa and provides a route. Insert the mini-forceps into the lymph node. Open and close the mini-forceps, then remove the mini-forceps. TBNA: transbronchial aspiration. Image credit: Yuki Takigawa.

EBUS-IFB is a technique in which forceps are passed into targeted peribronchial lymph nodes following EBUS-TBNA (Figure 4). Aspiration creates a hole (more than 1 mm) in the airway mucosa and provides a route for the insertion of forceps into the lymph node. EBUS-IFB was first reported in 2008 by Herth [7]; moreover, Agrawal et al. reported in a meta-analysis that the addition of EBUS-IFB to EBUS-TBNA improved the overall diagnostic yield of sampling intrathoracic adenopathy compared to that of EBUS-TBNA alone [3]. Regarding lymphoma, a total of five studies were analyzed, which revealed a yield of 30% (15 of 50) for EBUS-TBNA and 86% (43 of 50) for combined EBUS-TBNA and EBUS-IFB. Nakai et al. reported a sample size of 1.29±0.93 mm^2^ by mini-forceps (0.96 mm) and 3.46±2.11 mm^2^ by 1.9 mm-forceps. EBUS-IFB may yield larger, better-quality tissues and a more detailed diagnosis. EBUS-IFB complication rates are relatively low (pneumothorax, 1%; bleeding, 0.8%; and pneumomediastinum, 1%) [8].

EBUS-IFB may mainly performed in #7 or #4R, but also paratracheal and subcarinal locations [9]. Radchenko et al. reported that the mean size was 21.9 mm in hilar and interlobar locations [10]. Once the technique is mastered, EBUS-IFB seems possible if the EBUS-bronchoscope can be contacted to the lesion of even in relatively peripheral airways.

In our case, a recurrence of lymphoma was suspected, and tissue obtained by EBUS-TBNA was not be sufficient for pathological diagnosis because we performed ROSE during EBUS-TBNA. No malignant findings were observed upon final histological examination using EBUS-TBNA. We performed EBUS-IFB and could diagnose the recurrence of follicular lymphoma. Although the lymph node was relatively smaller and in the hilar location, we were able to approach #12 lymph node safety via EBUS-IFB.

## Conclusions

Currently, few cases have been reported on EBUS-IFB use in Japan. EBUS-IFB, which is a safety method for bronchoscopic biopsy of mediastinal lymph nodes, may enhances the diagnostic yield for lymphoma; however, if the target lesion is located in lymph node #12.
